# Combining non-negative matrix factorization with graph Laplacian regularization for predicting drug-miRNA associations based on multi-source information fusion

**DOI:** 10.3389/fphar.2023.1132012

**Published:** 2023-02-02

**Authors:** Mei-Neng Wang, Yu Li, Li-Lan Lei, De-Wu Ding, Xue-Jun Xie

**Affiliations:** ^1^ School of Mathematics and Computer Science, Yichun University, Yichun, China; ^2^ School of Information Engineering, Inner Mongolia University of Science and Technology, Baotou, China

**Keywords:** non-negative matrix factorization, graph Laplacian regularization, drug-miRNA associations, weighted k nearest neighbor, sparse similarity matrix

## Abstract

Increasing evidences suggest that miRNAs play a key role in the occurrence and progression of many complex human diseases. Therefore, targeting dysregulated miRNAs with small molecule drugs in the clinical has become a new treatment. Nevertheless, it is high cost and time-consuming for identifying miRNAs-targeted with drugs by biological experiments. Thus, more reliable computational method for identification associations of drugs with miRNAs urgently need to be developed. In this study, we proposed an efficient method, called GNMFDMA, to predict potential associations of drug with miRNA by combining graph Laplacian regularization with non-negative matrix factorization. We first calculated the overall similarity matrices of drugs and miRNAs according to the collected different biological information. Subsequently, the new drug-miRNA association adjacency matrix was reformulated based on the K nearest neighbor profiles so as to put right the false negative associations. Finally, graph Laplacian regularization collaborative non-negative matrix factorization was used to calculate the association scores of drugs with miRNAs. In the cross validation, GNMFDMA obtains AUC of 0.9193, which outperformed the existing methods. In addition, case studies on three common drugs (i.e., 5-Aza-CdR, 5-FU and Gemcitabine), 30, 31 and 34 of the top-50 associations inferred by GNMFDMA were verified. These results reveal that GNMFDMA is a reliable and efficient computational approach for identifying the potential drug-miRNA associations.

## 1 Introduction

Most of the human genes can be transcribed, but more than 98% of genes cannot encode proteins (only about 1.5% can encode proteins) (Carninci et al., 2005). In human tissues, some non-coding RNAs (ncRNAs) can regulate life activities by affecting genes and epigenetics. According to whether the length of ncRNA exceeds 200 nucleotides (nts), ncRNAs can be divided into long non-coding RNA (lncRNA) and short non-coding RNA (sncRNA) (Batista and Chang, 2013). MicroRNA (miRNA) is a type of small ncRNAs with about 22nts that is widely discovered in human beings, animals and plants (Wheeler et al., 2013). miRNAs perform post-transcriptional gene regulation by silencing gene expression (He and Hannon, 2004). Since the discovery of the first miRNA in 1993 in the Caenorhabditis elegans, more and more miRNAs have been discovered in various organisms (Wightman et al., 1993). Up to now, based on the recently updated miRBase (v22), there are 2,656 miRNAs reported and annotated in human beings (Kozomara et al., 2018). MiRNAs are not only highly conserved among different species, but also have temporal and tissue specificity in expression (Berezikov et al., 2006). In human tissues, More than 30% of human genes can be up-regulated or down-regulated by miRNA, and the number of target genes regulated by one miRNA even exceeds 200 (Sui et al., 2013). Research evidences suggest that miRNAs are widely participated in physiological processes and pathological, such as cell development, differentiation, proliferation and apoptosis, etc., (Bartel, 2004; You et al., 2017; Wang et al., 2019a). Clinical studies have confirmed that the occurrence and development of many complex diseases are closely related to the abnormal expression of some specific miRNAs, including tumor, neurological disorders, immune-related and cardiovascular (Rupaimoole and Slack, 2017; You et al., 2017; Peng et al., 2022). For example, the expression levels of miR-210, miR-221 and let-7d are up-regulated in invasive carcinoma and down-regulated in ductal carcinoma *in situ* (Di Leva et al., 2015). The expression of miR-21 is obviously up-regulated in liver cancer, breast cancer and other malignant tumors. MiR-21 negatively regulates the expression of the tumor suppressor gene PTEN to enhance the invasion and proliferation of liver cancer cells. Therefore, miRNAs have attracted increasing attention of researchers as diagnostic biomarkers and potential therapeutic targets for complex human diseases.

Small molecule drug is an organic compound with a small molecular weight (less than 1,000 Daltons) (Dougherty and Pucci, 2011). Most drugs are small molecule, among commonly used drugs, the number of small molecule drugs accounts for about 98% of the total (Krzyzosiak et al., 2018). Because of good drug-forming properties and drug metabolism, small molecule drugs are helpful to regulate biological processes (Krzyzosiak et al., 2018). Currently, proteins are as the main targets of drug in clinical medical treatment (Hopkins and Groom, 2002; Huang et al., 2018). However, only 10%–15% of human proteins with expression functions are thought to be associated with diseases (Dixon and Stockwell, 2009). In addition, among these disease-associated proteins, many molecules cannot be combined with drugs due to the lack of unique structures, which means that they cannot be targeted (Dixon and Stockwell, 2009; Wang et al., 2018). In other words, the number of protein-targeted of drugs is still very limited. Existing drugs actually only target about 0.05% of the human genome (Santos et al., 2017). In recent years, scientists have begun to look for new drug targets, such as lncRNA and miRNA. The number of targets will become very plentiful if lncRNAs and miRNAs can be as targets for drugs. Nowadays, studies have discovered that miRNAs can be targeted by drugs and have received increasing attentions (Jiang et al., 2012; Huang et al., 2021). Jiang et al. constructed a correlation diagram between drugs and miRNAs in human cancers, and confirmed that some of miRNAs can be inhibited by drugs (Jiang et al., 2012). For example, clinical trials have confirmed that SPC349 can inhibit miR-122 in hepatitis C viruses (Lanford et al., 2010). Additionally, in the breast cancer MCF-7 cells, the expression of miR-21 can be reduced by the use of Matrine (Li et al., 2012). Therefore, in-depth study of drug-miRNA associations is not only conducive to the discovery of new drugs, but also to the repositioning and resistance researches of existing drugs (Huang et al., 2020; Shen et al., 2022). Since the identification of drug-miRNA associations through biological experiments is time-consuming and costly, more accurate and efficient computational approaches for revealing their associations are imperative.

Based on the assumption that similar drugs tend to be related with similar miRNAs, some computational methods have been proposed to identify drug-miRNA associations, including Random Walk with Restart algorithm, Rotation Forest, and Graph Representation Learning, etc., Lv et al. developed a novel computational model to comprehensively infer the unknown associations of drug with miRNA by using Random Walk with Restart algorithm on the bipartite network (Lv et al., 2015). Guan et al. proposed a computational method of Graphlet Interaction based inference for drug-MiRNA association (GISMMA) (Guan et al., 2018). This method used Graphlet Interaction consisting of 28 isomers to describe the complex associations between two drugs or two miRNAs. The drug-miRNA association score is calculated by counting the numbers of graphlet interaction in miRNA similarity network and drug similarity network. Li et al*.* developed a new computational model based on network framework to infer miRNAs as potential biomarkers of anticancer drugs (SMiR-NBI) (Li et al., 2016a). This method implemented a network-based algorithm by constructing a heterogeneous network that connected genes, miRNAs and drugs. Yin et al*.* developed a computational approach using heterogeneous graph inference and sparse learning to discover associations of drug with miRNA (SLHGISMMA) (Yin et al., 2019). SLHGISMMA decomposes the adjacency matrix of drug-miRNA using sparse learning, and reconstructs heterogeneous graph for predicting. Qu et al. developed a triple layer heterogeneous graph method to discover drug-miRNA potential relationships (TLHNSMMA) (Qu et al., 2018). This method used an iterative update algorithm to transmit information through the constructed heterogeneous network. Wang et al*.* proposed a new computational model based on random forest (RFSMMA) (Wang et al., 2019b). The model of RFSMMA uses machine learning algorithms to infer drug-miRNA associations by integrating multiple similarities between drugs and miRNAs. Although many calculation methods have been proposed, as of now, these existing methods are still unsatisfactory for predicting drug-miRNA associations. In fact, drug-miRNA associations inference can be regarded as a recommender task (Huang et al., 2017; Wang et al., 2022a). Recent studies suggest that non-negative matrix factorization (NMF) has been effectively used for data representation in recommendation systems (Lee and Seung, 1999; Jiang et al., 2015), especially in the field of bioinformatics (Wang et al., 2021a; Wang et al., 2021b). Therefore, we turn the drug-miRNA association prediction into recommender system task and utilize NMF to discover potential associations between them.

In this work, we propose a new approach, GNMFDMA, to infer drug-miRNA potential associations by combining graph Laplacian regularization with non-negative matrix factorization. In our method, the similarity of drug needs to be measured by combining drug chemical structure similarity, drug side effect similarity, disease-phenotype similarity and gene-functional consistency similarity. The similarity of miRNA was measured by merging disease-phenotype and gene-functional consistency. In addition, we constructed the graph space of drug and miRNA using K -nearest neighbors, which guides the matrix factorization process so that similar drugs (miRNAs) are sufficiently close in the graph space (Cai et al., 2010; Huang et al., 2016). We performed five-fold cross-validation to assess the performance of GNMFDMA, and compared it with SMiR-NBI (Li et al., 2016a), SLHGISMMA (Yin et al., 2019), TLHNSMMA (Qu et al., 2018) and RFSMMA (Wang et al., 2019b). The experiment results demonstrated that the proposed method of GNMFDMA outperformed other methods of comparison. In the case studies for three common drugs, 5-Aza-CdR, 5-FU and Gemcitabine, 30, 31 and 34 out of the top 50 associations inferred were verified by experimental literatures, respectively. These results further suggest that GNMFDMA is an efficient model in revealing drug-miRNA potential associations.

## 2 Materials and methods

### 2.1 Methods overview

**FIGURE 1:**
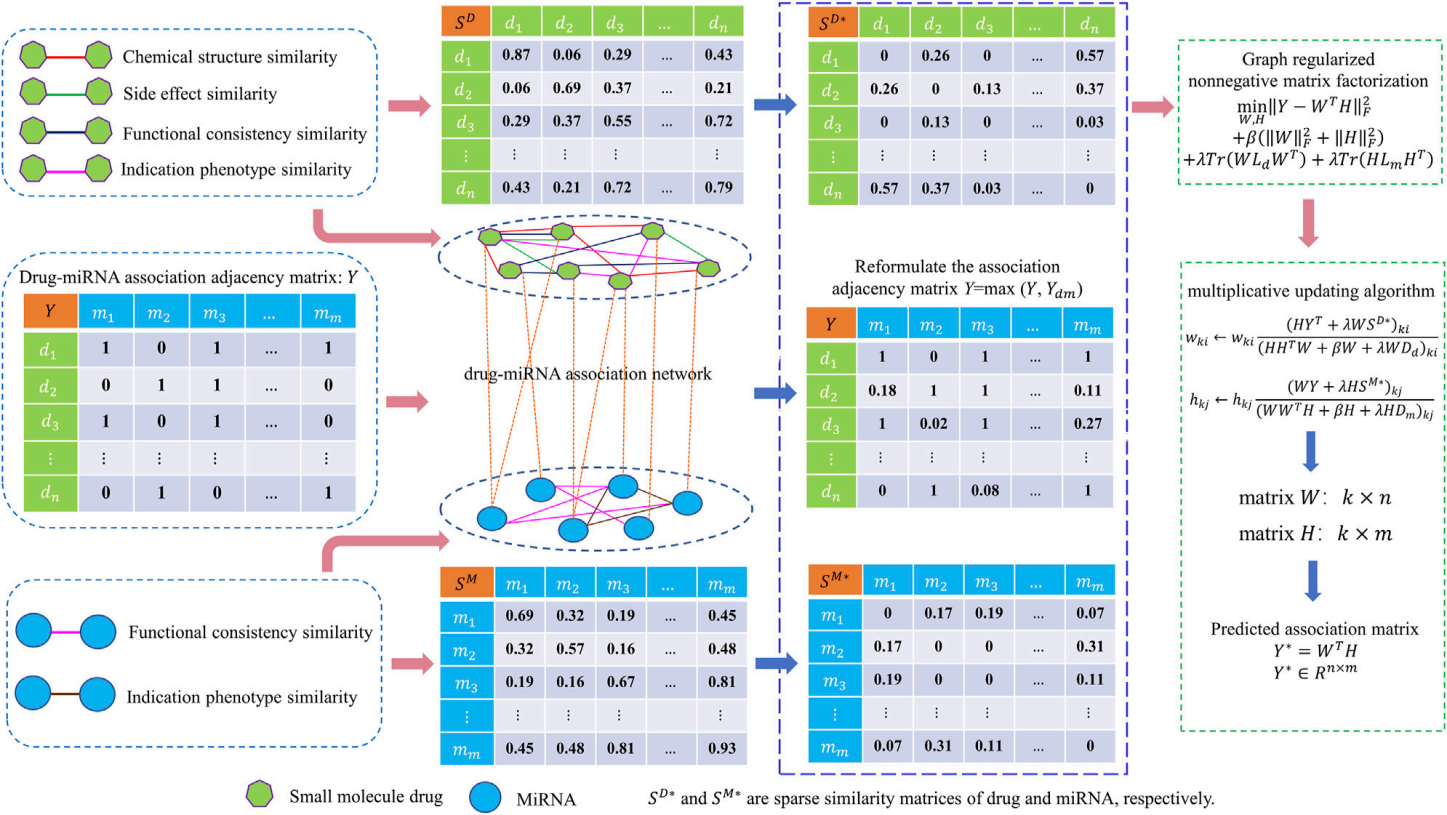
Overall framework of GNMFDMA for inferring potential drug-miRNA associations.

In this work, a new computational model called GNMFDMA is developed to predict associations of drug with miRNA. The GNMFDMA approach can be summarized into the following three steps (See Figure 1). First, the similarity matrix of drugs is constructed according to the drug chemical structure similarity, indication phenotype similarity of drug, drug side effect similarity and gene functional consistency similarity of drug. The similarity matrix of miRNAs is constructed based on gene functional consistency and disease indication phenotype similarity of miRNA. Second, to extend GNMFDMA to novel drugs and miRNAs, we use weighted K nearest neighbor profiles to re-construct the drug-miRNA association adjacency matrix. Finally, graph Laplacian regularization collaborative standard non-negative matrix factorization is utilized to discover drug-miRNA potential associations.

### 2.2 Construct the similarity networks of drug and miRNA

In order to infer potential associations of drug with miRNA using non-negative matrix factorization, we construct the drug-drug interaction network and miRNA-miRNA interaction network by integrating the four categories of drug-drug similarities and two categories of miRNA-miRNA similarities, respectively. Besides, drug-miRNA association network is constructed using the known drug-miRNA association pairs.

In this work, the verified 664 drug-miRNA associations were obtained from the SM2miR database, which can be accessible at http://bioinfo.hrbmu.edu.cn/SM2miR/ (Liu et al., 2012). In the 664 known associations, 831 drugs obtain from PubChem (Wang et al., 2009), DrugBank (Knox et al., 2010), and SM2miR; 541 miRNAs collect from PhenomiR (Ruepp et al., 2010), HMDD (Lu et al., 2008), miR2Disisease (Jiang et al., 2008), and SM2miR databases. However, there are some drugs and miRNAs without any known association information. For this reason, these drugs and miRNAs are deleted, and the duplicated entries are also removed. After screening, the drug-miRNA association network with 664 different associations is constructed for prediction, including 39 drugs and 286 miRNAs (See Table 1). Based on the drug-miRNA association network, the original association adjacency matrix $Y\in R^{n\times m}$ is defined, where m and n represent the number of miRNAs and drugs, respectively. The element value $Y\left(i,j\right)$ is set to one if drug $d\left(i\right)$ is confirmed to be associated with miRNA $m\left(j\right)$ , otherwise it is 0.

**TABLE 1:** The dataset used in GNMFDMA for prediction.

Dataset	Drugs	MiRNAs	Associations	Sparsity (%)
	39	286	664	5.59

Previous studies have shown that similarities based on chemical structure (Hattori et al., 2003), indication phenotype (Gottlieb et al., 2011), side effect (Gottlieb et al., 2011) and gene functional consistency (Lv et al., 2011) are effectively tools to infer the relationships between drugs. In this work, to avoid the bias of single similarity measurement and contribute the discovery of new interactions, four types of drug similarity were integrated according to the model of Lv et al. (2015). The four types of drug similarity are drug chemical structure similarity, disease indication phenotype similarity of drug, drug side effect similarity and gene functional consistency similarity of drug, respectively (Lv et al., 2015). We use matrix $S_{g}^{d}$ to represent the drug similarity information based on gene functional consistency. The element $S_{g}^{d}\left(i,j\right)$ of matrix $S_{g}^{d}$ is the functional consistency similarity of drug $d\left(i\right)$ and drug $d\left(j\right)$ . At the same time, $S_{c}^{d}$ , $S_{s}^{d}$ and $S_{d}^{d}$ denote the similarity matrices based on chemical structure, side effect and disease indication phenotype, respectively. For each pair of drugs, four types of similarity are combined to calculate the overall similarity as follows:

$$S^{D}=\frac{{\omega_{1}S}_{c}^{d}+\omega_{2}S_{d}^{d}+\omega_{3}S_{g}^{d}+\omega_{4}S_{s}^{d}}{\omega_{1}+\omega_{2}+\omega_{3}+\omega_{4}}$$ (1)

where the weight value $\omega_{1},\omega_{2},\omega_{3}$ and $\omega_{4}$ are assigned as 1, respectively. The size of $S^{D}$ is n×n , The element $S^{D}\left(i,j\right)$ denotes the similarity of drug $d\left(i\right)$ with drug $d\left(j\right)$ .

The similarity of miRNA is constructed in this work using the model proposed by Lv et al. (2015), which is based on disease indication phenotype similarity of miRNA and gene functional consistency similarity of miRNA, respectively (Gottlieb et al., 2011; Lv et al., 2011). $S_{d}^{m}$ and $S_{g}^{m}$ denote disease indication phenotype similarity of miRNA and gene functional consistency similarity of miRNA. Then, we calculate the overall similarity of miRNA by integrating the two types of similarity $S_{g}^{m}$ and $S_{d}^{m}$ as follows:

$$S^{M}=\frac{\sigma_{1}S_{d}^{m}+\sigma_{2}S_{g}^{m}}{\sigma_{1}+\sigma_{2}}$$ (2)

where the weight value $\sigma_{1}$ and $\sigma_{2}$ are assigned as 1, respectively. The size of $S^{M}$ is m×m , the element $S^{M}\left(i,j\right)$ is the similarity of miRNA $m\left(i\right)$ with miRNA $m\left(j\right)$ .

### 2.3 Weighted K nearest known neighbors (WKNKN)

Let $D=\left\{d_{1},d_{2},\cdots ,d_{n}\right\}$ and $M=\left\{m_{1},m_{2},\cdots ,m_{m}\right\}$ are the set of n drugs and m miRNAs. The ith row vector $Y\left(d_{i}\right)=\left(Y_{i1},Y_{i2},\cdots ,Y_{im}\right)$ and the jth column vector $Y\left(m_{j}\right)=\left(Y_{1j},Y_{2j},\cdots ,Y_{nj}\right)$ of matrix Y denote the interaction profiles of drug $d_{i}$ and miRNA $m_{j}$ , respectively. For a novel drug without any known associated miRNAs or a novel miRNA without any known associated drugs, there are no interactions in their profiles. In fact, many of unknown drug-miRNA association pairs (or 0’s) in Y could be potential true associations, which may result in a higher false positive rate and reduce prediction performance. In order to address this problem, a preprocessing step (WKNKN) is performed to construct new interaction profiles based on their known neighbors.

For each drug $d_{l}$ , all other drugs are ranked in descending order on the basis of their similarity to $d_{l}$ . Then, the new interaction profile for drug $d_{l}$ is obtained based on their corresponding interaction profiles of the *K* known drugs nearest to $d_{l}$ (Ezzat et al., 2017):

$$Y_{d}\left(d_{l}\right)=\frac{1}{\sum_{1\leq i\leq K}S^{D}\left(d_{i},d_{l}\right)}\overset{K}{\underset{i=1}{\sum }}\theta_{i}Y\left(d_{i}\right)$$ (3)

where $\theta_{i}=\alpha^{i-1}*S^{D}\left(d_{i},d_{l}\right)$ is the weight coefficient, a larger $\theta_{i}$ represents that $d_{i}$ and $d_{l}$ are more similar. $\alpha \in \left[0,1\right]$ is a decay term. The same procedure for miRNA, for each miRNA $m_{p}$ , the new interaction profile can be defined as follows:

$$Y_{m}\left(m_{p}\right)=\frac{1}{\sum_{1\leq j\leq K}S^{M}\left(m_{j},m_{p}\right)}\overset{K}{\underset{j=1}{\sum }}\theta_{j}Y\left(m_{j}\right)$$ (4)

Similarly, all other miRNAs are ranked in descending order according to their similarity to $m_{p}$ . $\theta_{j}=\alpha^{j-1}*S^{M}\left(m_{j},m_{p}\right)$ is the weight coefficient.

Then, we merge the two matrices of $Y_{d}$ and $Y_{m}$ , and replace $Y_{ij}=0$ with the associated likelihood score. Finally, the novel drug-miRNA association adjacency matrix is obtained:

$$Y=\max\;\left(Y,\;Y_{dm}\right)$$ (5)

where

$$Y_{dm}=\frac{Y_{d}+Y_{m}}{2}$$ (6)

### 2.4 Sparse similarity matrices

Based on the spectral graph and manifold learning theories that the nearest neighbor graph can maintain the local geometry of the original data points, and the sparseness technique of similarity matrix has been successfully applied in graph regularization (Cai et al., 2010; You et al., 2010; Li et al., 2016b). At the same time, the drugs and miRNAs located in the same cluster often have more similar functions. Thus, we calculate the affinity graphs ( $S^{D*}$ ; $S^{M*}$ ) for drug space and miRNA space using p -nearest neighbor. Then, the weight matrix of drug is defined according to the drug similarity matrix $S^{D}$ as follows:

$$G_{ij}^{D}=\left\{\begin{array}{c} \;1\;i\in N_{p}\left(d_{j}\right)\&j\in N_{p}\left(d_{i}\right)\\ 0\;i\notin N_{p}\left(d_{j}\right)\&j\notin N_{p}\left(d_{i}\right)\\ 0.5\;otherwise\; \end{array}\right.$$ (7)

where $N_{p}\left(d_{i}\right)$ and $N_{p}\left(d_{j}\right)$ are the sets of *p*-nearest neighbors of drug $d_{i}$ and drug $d_{j}$ , respectively. Finally, the sparse similarity matrix $S^{D*}$ for drugs is calculated as:

$$\forall i,j,\;S_{ij}^{D*}=S_{ij}^{D}G_{ij}^{D}$$ (8)

Similarly, the sparse similarity matrix $S^{M*}$ for miRNAs is calculated as follows:

$$\forall i,j,\;S_{ij}^{M*}=S_{ij}^{M}G_{ij}^{M}$$ (9)

### 2.5 The model of GNMFDMA

Non-negative matrix factorization (NMF) method has been effectively applied for data representation. NMF decomposes an original matrix into two non-negative matrices whose product is as equal to the original matrix as possible. At the same time, it can also achieve the purpose of dimensionality reduction. In this work, NMF is used to decompose the drug-miRNA association adjacency matrix $Y^{n\times m}$ into $W^{k\times n}$ ; $H^{k\times m}$ ( $k<\min\left(m,\;n\right)$ ), and $Y≅W^{T}H$ . The problem of drug-miRNA association prediction can be expressed by the following objective function:

$$\min_{W,H}{\left\|Y-W^{T}H\right\|}_{F}^{2}\;s,t.\;W\geq 0,\;H\geq 0$$ (10)

where ${\left\|∙\right\|}_{F}^{2}$ is the Frobenius norm and k is the subspace dimensionality. However, in the Euclidean space, the intrinsic geometrical of the drug or miRNA space cannot be discovered by standard NMF (Wang et al., 2022b). To prevent overfitting and enhance generalization capability of the model, the graph Laplacian regularization terms and Frobenius norm regularization terms (Tikhonov $L_{2}$ ) are introduced to the standard NMF. The graph Laplacian regularization can ensure local invariance for data space (Cai et al., 2010). Here, we use graph Laplacian regularization to ensure close miRNAs or drugs to be adequately close to each other in miRNA or drug corresponding space. In addition, the Frobenius norm regularization terms are utilized to guarantee the smoothness of W and H . Therefore, the objective function of GNMFDMA can be transformed into:

$$\min_{W,H}{\left\|Y-W^{T}H\right\|}_{F}^{2}+\lambda \left(\sum_{i\leq j=1}^{n}{\left\|w_{i}-w_{j}\right\|}^{2}S_{ij}^{D*}+\sum_{i\leq j=1}^{m}{\left\|h_{i}-h_{j}\right\|}^{2}S_{ij}^{M*}\;\right)+\beta \left({\left\|W\right\|}_{F}^{2}+{\left\|H\right\|}_{F}^{2}\right)\;\;\;\;s,t.\;W\geq 0,\;H\geq 0$$ (11)

where β and λ represent the sparseness constraint coefficient and regularization coefficient, respectively. $w_{i}$ and $h_{j}$ are the ith and jth columns of W and H , respectively.

$$R_{d}=\sum_{i\leq j=1}^{n}{\left\|w_{i}-w_{j}\right\|}^{2}S_{ij}^{D*}$$

$$=\overset{n}{\underset{j=1}{\sum }}w_{j}^{T}w_{j}\overset{n}{\underset{i,j=1}{\sum }}S_{ij}^{D*}-\overset{n}{\underset{i,j=1}{\sum }}w_{i}^{T}w_{j}S_{ij}^{D*}$$

$$=\overset{r}{\underset{j=1}{\sum }}w_{j}^{T}w_{j}D_{jj}-\overset{r}{\underset{i,j=1}{\sum }}w_{i}^{T}w_{j}S_{ij}^{D*}$$

$$=Tr\left(WD_{d}W^{T}\right)-Tr\left(WS^{D*}W^{T}\right)=Tr\left(WL_{d}W^{T}\right)$$ (12)

and

$$R_{m}=\sum_{i\leq j=1}^{m}{\left\|h_{i}-h_{j}\right\|}^{2}S_{ij}^{M*}=Tr\left(HL_{m}H^{T}\right)$$ (13)

here, $Tr\left(∙\right)$ denotes the trace of matrix. $R_{d}$ and $R_{m}$ are the graph Laplacian regularization terms. $L_{d}=D_{d}-S^{D*}$ is graph Laplacian matrix of $S^{D*}$ , $L_{m}=D_{m}-S^{M*}$ is graph Laplacian matrix of $S^{M*}$ , respectively (Liu et al., 2014). $D_{d}$ and $D_{m}$ are the diagonal matrices, $D_{d}\left(i,i\right)=\sum_{l=1}^{n}S_{il}^{D*}$ and $D_{m}\left(j,j\right)=\sum_{p=1}^{m}S_{jp}^{M*}$ . Eq. 11 can be expressed as follows:

$$\begin{array}{c} \min_{W,H}{\left\|Y-W^{T}H\right\|}_{F}^{2}+\beta \left({\left\|W\right\|}_{F}^{2}+{\left\|H\right\|}_{F}^{2}\right)+\lambda Tr\left(WL_{d}W^{T}\right)+\lambda Tr(HL_{m}H^{T})=Tr(YY^{T})+{Tr(W}^{T}HH^{T}W)\\ -2Tr(YH^{T}W)+\beta Tr\left(W^{T}W\right)+\beta Tr\left(H^{T}H\right)+\lambda Tr\left(WL_{d}W^{T}\right)+\lambda Tr\left(HL_{m}H^{T}\right) \end{array}$$ (14)

### 2.6 Optimization of GNMFDMA

To minimize Eq. 14, we introduce Lagrange multipliers method to solve this problem. Let Lagrange multipliers $\psi =\left\{\varphi_{ki}\right\}$ and $\Phi =\left\{\phi_{kj}\right\}$ to ensure $w_{ki}\geq 0$ and $h_{kj}\geq 0$ . The corresponding optimization function F of Eq. 14 is formularized as:

$$\begin{array}{ll} F & =Tr(YY^{T})+{Tr(W}^{T}HH^{T}W)-2Tr(YH^{T}W)+\beta Tr(W^{T}W)+\beta Tr\left(H^{T}H\right)\\ & +\lambda Tr\left(WL_{d}W^{T}\right)+\lambda Tr\left(HL_{m}H^{T}\right)+\psi Tr\left(W^{T}\right)+\Phi Tr\left(H^{T}\right) \end{array}$$ (15)

The partial derivatives of F for W and H are:

$$\frac{\partial F}{\partial W}=2HH^{T}W-2HY^{T}+2\beta W+2\lambda WL_{s}+\psi$$ (16)

$$\frac{\partial F}{\partial H}=2WW^{T}H-2WY+2\beta H+2\lambda HL_{m}+\Phi$$ (17)

Then, the Karush–Kuhn–Tucker (KKT) condition $\varphi_{ki}w_{ki}=0$ ; $\phi_{kj}h_{kj}=0$ are used in Eq. 16 and Eq. 17 (Facchinei et al., 2014). We can obtain the following Equations:

$${\left(HH^{T}W\right)}_{ki}w_{ki}-{\left(HY^{T}\right)}_{ki}w_{ki}+{\left(\beta W\right)}_{ki}w_{ki}+{\left[\lambda W\left(D_{d}-S^{D*}\right)\right]}_{ki}w_{ki}=0$$ (18)

$${\left(WW^{T}H\right)}_{kj}h_{kj}-{\left(WY\right)}_{kj}h_{kj}+{\left(\beta H\right)}_{kj}h_{kj}+{\left[\lambda H\left(D_{m}-S^{M*}\right)\right]}_{kj}h_{kj}=0$$ (19)

Thus, the updating rules for $w_{ki}$ and $h_{kj}$ can be obtained as follows:

$$w_{ki}\leftarrow w_{ki}\frac{{\left(HY^{T}+\lambda WS^{D*}\right)}_{ki}}{{\left(HH^{T}W+\beta W+\lambda WD_{d}\right)}_{ki}}$$ (20)

$$h_{kj}\leftarrow h_{kj}\frac{{\left(WY+\lambda HS^{M*}\right)}_{kj}}{{\left(WW^{T}H+\beta H+\lambda HD_{m}\right)}_{kj}}$$ (21)

Updating $w_{ki}$ and $h_{kj}$ with Eq. 20 and Eq. 21 until W and H reach the following convergence conditions:

$$\forall i,\;{\left\|w_{i}^{\left(l+1\right)}-w_{i}^{\left(l\right)}\right\|}_{F}^{2}\leq 10^{-4}$$ (22)

$$\forall j,\;{\left\|h_{j}^{\left(l+1\right)}-h_{j}^{\left(l\right)}\right\|}_{F}^{2}\leq 10^{-4}$$ (23)

Ultimately, the predicted drug-miRNA associations adjacency matrix $Y^{*}$ is calculated by $Y^{*}=W^{T}H$ . The elements of matrix $Y^{*}$ are regarded as the drug-miRNA association predicted scores. For each drug-miRNA pair, all the miRNAs are sorted in descending order based on the predicted scores. In theory, the top ranked miRNAs in predicted matrix $Y^{*}$ are more possible to be related to the corresponding drug.

## 3 Results

### 3.1 Experimental settings

To systematical evaluate the performance of GNMFDMA, we carry out five-fold cross-validation (5-CV) experiments on SM2miR database and compare it with four state-of-the-art predictors: SMiR-NBI (Li et al., 2016a), SLHGISMMA (Yin et al., 2019), TLHNSMMA (Qu et al., 2018) and RFSMMA (Wang et al., 2019b). Specifically, in the framework of five-fold cross-validation, 664 known drug-miRNA association pairs are randomly divided into five equal subsets. Four subsets of them are taken in turn as the training samples to train the prediction model, and the remaining one subset is regarded as the test sample. In this work, the AUC values (the area under the ROC curve) are used to assess the prediction performance of various models. AUC = 0.5 represents randomly prediction, whereas AUC = 1 represents that the prediction performance of the method is perfect.

In this paper, the parameter values are chosen by 5-CV experiment on the training dataset. GNMFDMA has the following five parameters, the neighborhood size K and decay value α are chosen from $\left\{1,\;2,\;3,\;4,\;5\right\}$ and $\left\{01,\;0.2,\;0.3,\cdots \cdots ,\;0.9,\;1\right\}$ when the adjacency matrix is reformulated, respectively. For non-negative matrix factorization, three parameters are subspace dimensionality k , regularization coefficient λ and sparseness constraint coefficient β , whose combinations are regarded from the following values: $k\in \left\{15,\;20,\;25,\;30,\;35\right\}$ , $\lambda \in \left\{0.2,\;0.6,\;1,\;2\right\}$ and $\beta \in \left\{0.002,\;0.02,\;0.2,\;0.6\right\}$ . According to previous studies (Cai et al., 2010), let p=5 when constructing the graph spaces for drug and miRNA. In order to more fairly comparison with previous methods, the parameters in other methods are all taken the optimal values recommended by authors. Finally, the parameters optimized values of our model are K=3 , α=0.9 , k=35 , λ=1 and β=0.02 .

### 3.2 Performance evaluation

**FIGURE 2:**
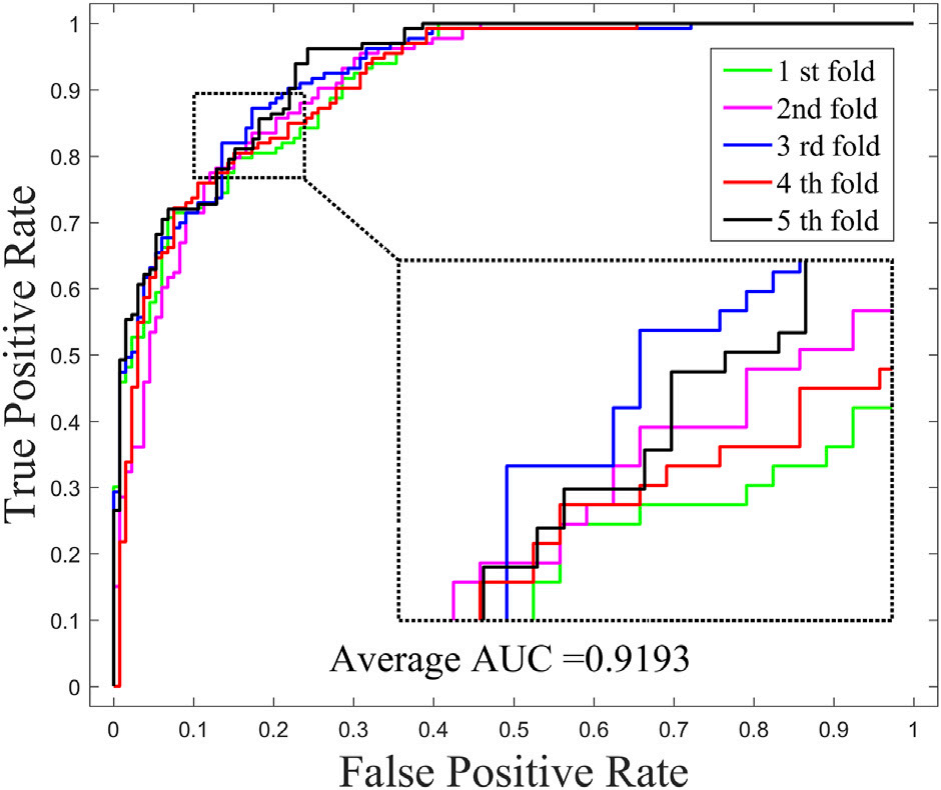
The ROC curves of GNMFDMA for drug-miRNA association prediction under 5-CV.

The performance of GNMFDMA is evaluated by comparing with the previous computational models: SMiR-NBI, SLHGISMMA, TLHNSMMA and RFSMMA. For the above methods, we all use 5-CV to evaluate their performance. Figure 2 draws the ROC curves of GNMFDMA, Table 2 displays the AUC values of all compared approaches. The AUC values of GNMFDMA, SMiR-NBI, SLHGISMMA, TLHNSMMA and RFSMMA are 0.9193, 0.7104, 0.7724, 0.8168 and 0.8389, respectively. GNMFDMA achieves the best performance, which are 20.89%, 14.69%, 10.25% and 8.04% higher than the other four computational methods, respectively.

**TABLE 2:** The AUC values of GNMFDMA and four compared methods in 5-CV.

Methods	GNMFDMA	SMiR-NBI	SLHGISMMA	TLHNSMMA	RFSMMA
AUC	0.9193	0.7104	0.7724	0.8168	0.8389

Additionally, in order to calculate the ratio of exact identifications in the predicted results, sensitivity (Sen), accuracy (Acc), precision (Pre) and F1-Score are widely applied to measure the model performance.

$$Sen.=\frac{TP}{TP+FN}$$ (24)

$$Pre.=\frac{TP}{TP+Fp}$$ (25)

$$Acc.=\frac{TN+TP}{TN+TP+FN+Fp}$$ (26)

$$F1-Score=\frac{2\times Pre.\times Sen.}{Pre.+Sen.}$$ (27)

Here, when given a cutoff value, TP and FP denote the number of true positive samples and false positive samples, whose prediction scores higher than cutoff value; TN and FN are the number of true negative samples and false negative samples, whose prediction scores lower than cutoff value. In this work, the threshold of specificity is set 85% to calculate sensitivity, accuracy, precision and F1-Score, respectively. Table 3 exhibits the sensitivity, accuracy, precision, and F1-Score by GNMFDMA under 5-CV.

**TABLE 3:** The average sensitivity, precision, accuracy and F1-Score obtained by GNMFDMA.

Fold	Sen.(%)	Pre.(%)	Acc.(%)	F1-score(%)	AUC
1	77.44	83.74	81.20	80.46	0.9155
2	78.20	83.87	81.58	80.93	0.9117
3	81.95	84.50	83.46	83.21	0.9252
4	77.44	83.74	81.20	80.46	0.9123
5	79.54	84.00	82.20	81.71	0.9230
**Average**	**78.91 ± 1.90**	**83.97 ± 0.32**	**81.93 ± 0.95**	**81.35 ± 1.16**	**0.9193 ± 0.0089**

The bold values represent the average and standard deviation for each column.

**FIGURE 3:**
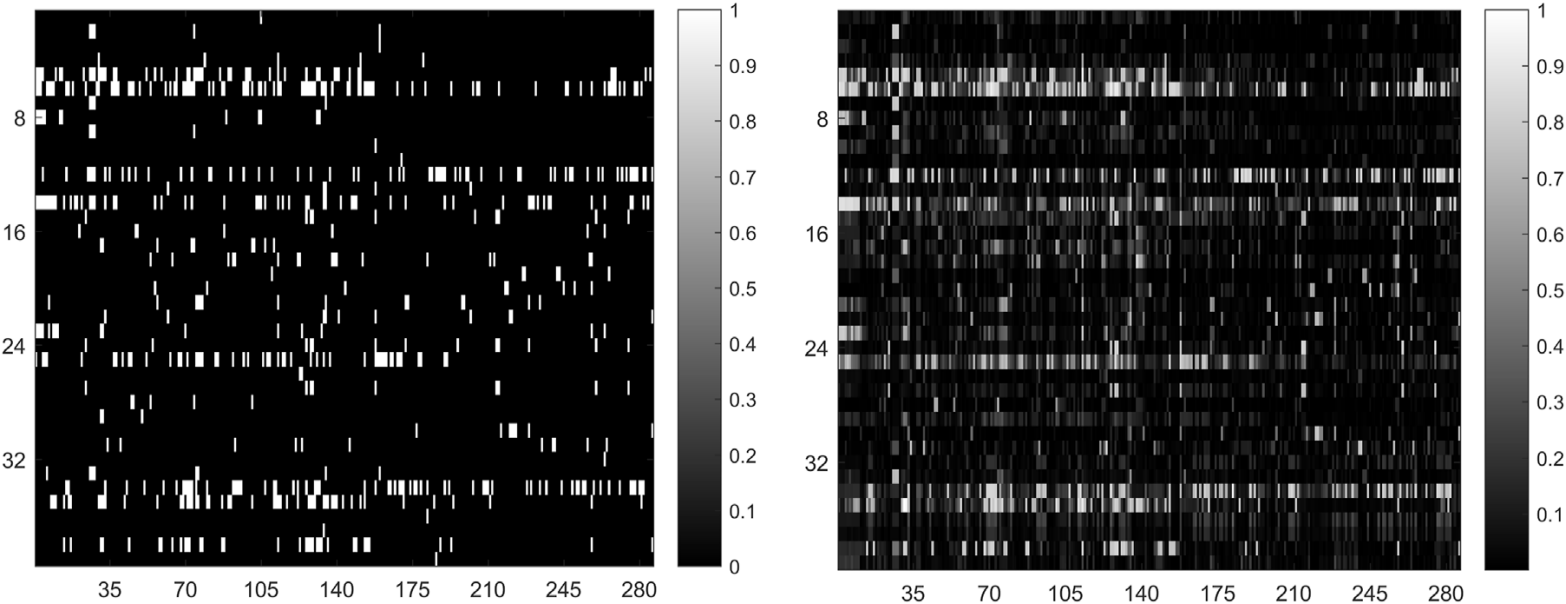
The comparison of original association adjacency matrix (left) and the prediction association matrix (right).

In general, the predicted results obtained from top-ranked are more convincing compared with those obtained from other portions. The more true association pairs that are correctly retrieved from the top-ranked, the predictor is more effective. For this reason, we calculate the correct recovery of association pairs at different thresholds when all 664 known drug-miRNA association pairs are used as training samples. The top 10%, 15% and 20% drug-related miRNAs in prediction result, GNMFDMA correctly retrieved 429 (64.61%), 532 (80.12%) and 617 (92.92%) association pairs, respectively. The comparison between the original association adjacency matrix and the predicted association matrix is shown in Figure 3. These results show that GNMFDMA can effectively retrieve the true association pairs with a lower false negative rate. In summary, the method of GNMFDMA has powerful ability for identifying drug-associated miRNAs.

### 3.3 The effect of WKNKN on model performance

**FIGURE 4:**
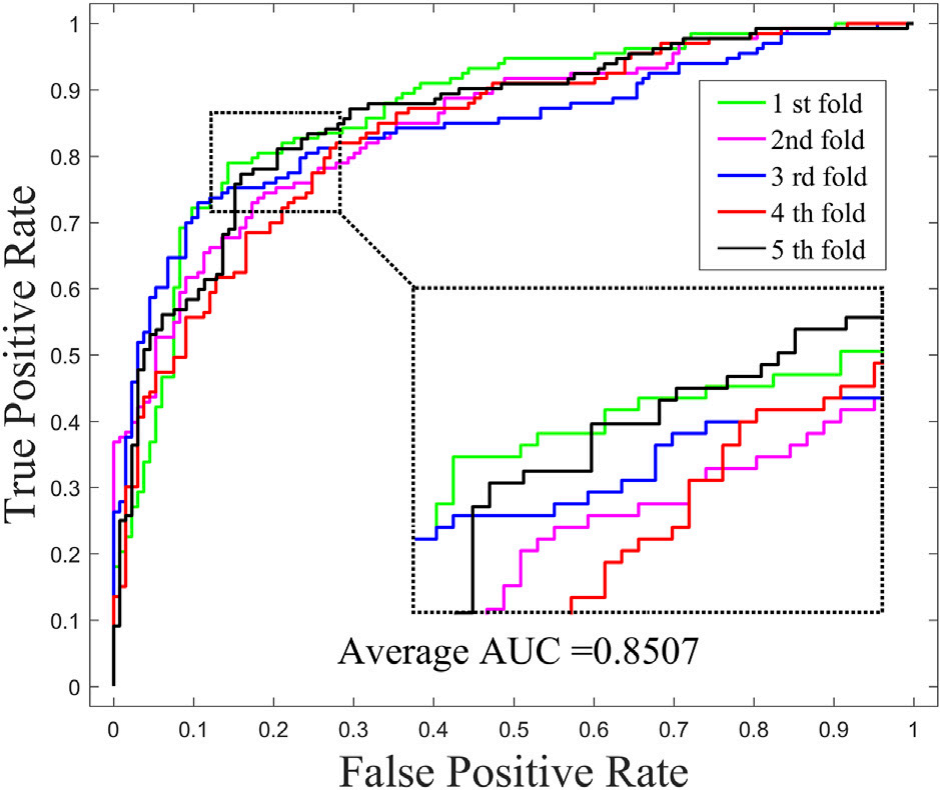
The ROC curves of GNMFDMA* for drug-miRNA association prediction under 5-CV.

In order to investigate the effects of preprocessing step (WKNKN) for GNMFDMA, we compared the performance of GNMFDMA and GNMFDMA* under 5-CV. For GNMFDMA, we implement a preprocessing step (WKNKN) to re-construct the drug-miRNA association adjacency matrix based on their known neighbors before performing non-negative matrix factorization, which can supplement more interaction information to give assistance for predicting new drugs and miRNAs. In addition, the preprocessing step is also helpful for predicting those drugs or miRNAs with sparse known associations. For GNMFDMA*, the preprocessing step is ignored and matrix factorization is directly performed on the original adjacency matrix for inferring drug-associated miRNAs. Figure 2 and Figure 4 represent the ROC curves of GNMFDMA and GNMFDMA* under 5-CV, the AUC values achieved by GNMFDMA and GNMFDMA* are 0.9193 and 0.8507, respectively. The results demonstrate that the performance of GNMFDMA is significantly improved after performing the preprocessing step.

### 3.4 Case studies

To further demonstrate the availability of GNMFDMA to discover potential associations of drug with miRNA, case studies are conducted for three common small molecule drugs, 5-Aza-CdR, 5-FU and Gemcitabine based on the SM2miR dataset. In each independent case study, all known 5-Aza-CdR (5-FU or Gemcitabine)-related miRNAs are removed (all miRNAs are regarded as the potential candidates of corresponding drug), the remaining known associations are utilized as the training samples. Next, for each investigated drug, these miRNAs are sorted in descending order according to the predicted scores, it means that the top-ranked miRNAs tend to be related to the corresponding drug.

We use the experimental literature to verify the predicted potential miRNAs for three corresponding drugs. The top 50 potential candidate miRNAs associated with 5-Aza-CdR, 5-FU and Gemcitabine predicted by GNMFDMA are exhibited in Table 4, Table 5 and Table 6, respectively. 30, 31 and 34 out of the top-50 miRNAs inferred by GNMFDMA are verified to be related to the corresponding drug by the experimental literature, respectively. For example, the expression of hsa-let-7d and hsa-let-7e was significantly down-regulated in gemcitabine-resistant cells (Li et al., 2009). Up-regulation of has-let-7 by natural agents can lead to the reversal of epithelial-to-mesenchymal transition in gemcitabine-resistant pancreatic cancer cells. Hsa-miR-125a promotes chemical resistance of pancreatic cancer cells to Gemcitabine by targeting A20 (Yao et al., 2016). In addition, the SM2miR database confirmed that hsa-miR-125a is also associated with drug 5-Aza-CdR. That is, one miRNA may be targeted by multiple small molecule drugs. The above results show that GNMFDMA can effectively predict new drugs or miRNAs without any known relationships, which has important reference significance for related biomedical experiments.

**TABLE 4:** The top-50 miRNAs related to 5-Aza-CdR are predicted by GNMFDMA.

Rank	MiRNA	Evidence	Rank	MiRNA	Evidence
1	hsa-mir-125b-1	26198104	26	hsa-mir-212	26693054
2	hsa-mir-18a	unconfirmed	27	hsa-mir-199a-2	30651148
3	hsa-mir-125b-2	26198104	28	hsa-mir-128–2	unconfirmed
4	hsa-mir-181a-2	26198104	29	hsa-mir-197	unconfirmed
5	hsa-mir-203a	26577858	30	hsa-mir-129–2	26081366
6	hsa-mir-19b-1	unconfirmed	31	hsa-mir-345	21665895
7	hsa-mir-19a	26198104	32	hsa-mir-181b-1	unconfirmed
8	hsa-mir-20a	26198104	33	hsa-mir-326	unconfirmed
9	hsa-mir-17	26198104	34	hsa-let-7a-1	unconfirmed
10	hsa-mir-181a-1	26198104	35	hsa-mir-329–1	unconfirmed
11	hsa-mir-324	unconfirmed	36	hsa-mir-133a-1	unconfirmed
12	hsa-mir-342	unconfirmed	37	hsa-mir-132	26675712
13	hsa-mir-320a	26198104	38	hsa-mir-187	unconfirmed
14	hsa-mir-328	23991164	39	hsa-mir-26a-1	unconfirmed
15	hsa-mir-16–1	26198104	40	hsa-mir-145	27364572
16	hsa-mir-155	26198104	41	hsa-mir-181b-2	unconfirmed
17	hsa-mir-27a	26198104	42	hsa-mir-217	24,350,829
18	hsa-mir-24–1	26198104	43	hsa-mir-202	unconfirmed
19	hsa-let-7c	24704393	44	hsa-mir-409	unconfirmed
20	hsa-mir-21	26198104	45	hsa-mir-125a	26198104
21	hsa-mir-27b	26198104	46	hsa-mir-372	unconfirmed
22	hsa-mir-339	unconfirmed	47	hsa-mir-373	21785829
23	hsa-let-7d	26802971	48	hsa-mir-186	30793488
24	hsa-let-7b	26708866	49	hsa-mir-211	unconfirmed
25	hsa-mir-154	26672991	50	hsa-mir-346	unconfirmed

*Note*: 5-Aza-CdR’s Compound ID (CID) in PubChem is 451668.

**TABLE 5:** The top-50 miRNAs related to 5-FU are predicted by GNMFDMA.

Rank	MiRNA	Evidence	Rank	MiRNA	Evidence
1	hsa-mir-324	30103475	26	hsa-mir-202	unconfirmed
2	hsa-mir-24–1	26198104	27	hsa-mir-132	26198104
3	hsa-mir-500a	unconfirmed	28	hsa-mir-299	31786874
4	hsa-mir-501	26198104	29	hsa-mir-326	26239225
5	hsa-mir-24–2	26198104	30	hsa-mir-181a-2	unconfirmed
6	hsa-mir-874	27221209	31	hsa-mir-1-2	unconfirmed
7	hsa-mir-650	unconfirmed	32	hsa-mir-154	unconfirmed
8	hsa-mir-23a	26198104	33	hsa-mir-27a	26198104
9	hsa-let-7b	25,789,066	34	hsa-mir-199a-2	26198104
10	hsa-mir-1226	26198104	35	hsa-mir-217	24255072
11	hsa-let-7c	2,5951903	36	hsa-mir-211	28720546
12	hsa-mir-155	28347920	37	hsa-mir-342	26198104
13	hsa-mir-21	26198104	38	hsa-mir-346	unconfirmed
14	hsa-mir-345	unconfirmed	39	hsa-mir-329–1	unconfirmed
15	hsa-mir-129–2	23744359	40	hsa-mir-149	26198104
16	hsa-let-7a-1	26198104	41	hsa-mir-339	unconfirmed
17	hsa-mir-181b-2	unconfirmed	42	hsa-mir-128–2	26198104
18	hsa-mir-194–1	unconfirmed	43	hsa-mir-133a-1	26198104
19	hsa-mir-409	unconfirmed	44	hsa-let-7d	26198104
20	hsa-mir-212	unconfirmed	45	hsa-mir-187	28347920
21	hsa-mir-26a-1	unconfirmed	46	hsa-mir-455	21743970
22	hsa-mir-197	26198104	47	hsa-mir-330	28521444
23	hsa-mir-205	24396484	48	hsa-mir-181a-1	unconfirmed
24	hsa-mir-337	unconfirmed	49	hsa-mir-128–1	26198104
25	hsa-mir-181b-1	unconfirmed	50	hsa-mir-329–2	unconfirmed

*Note*: 5-FU’s Compound ID (CID) in PubChem is 3,385.

**TABLE 6:** The top-50 miRNAs related to Gemcitabine are predicted by GNMFDMA.

Rank	MiRNA	Evidence	Rank	MiRNA	Evidence
1	hsa-mir-24–2	25841339	26	hsa-mir-15a	unconfirmed
2	hsa-mir-24–1	26198104	27	hsa-let-7a-2	23335963
3	hsa-mir-23a	unconfirmed	28	hsa-let-7a-3	23335963
4	hsa-mir-501	unconfirmed	29	hsa-mir-106b	31374207
5	hsa-mir-1226	unconfirmed	30	hsa-mir-16–2	unconfirmed
6	hsa-mir-500a	unconfirmed	31	hsa-let-7e	19654291
7	hsa-mir-324	26198104	32	hsa-mir-342	26198104
8	hsa-mir-650	unconfirmed	33	hsa-mir-210	31713003
9	hsa-mir-27b	25184537	34	hsa-mir-18a	28822990
10	hsa-mir-874	unconfirmed	35	hsa-mir-455	unconfirmed
11	hsa-mir-27a	26198104	36	hsa-mir-125a	26758190
12	hsa-let-7f-1	19948396	37	hsa-mir-93	unconfirmed
13	hsa-let-7d	26198104	38	hsa-mir-133a-1	26198104
14	hsa-mir-17	unconfirmed	39	hsa-mir-128–2	26198104
15	hsa-let-7g	19948396	40	hsa-mir-10a	24040438
16	hsa-mir-320a	26198104	41	hsa-mir-25	24040438
17	hsa-mir-20a	24924176	42	hsa-mir-197	26198104
18	hsa-let-7a-1	26198104	43	hsa-mir-149	26198104
19	hsa-mir-191	unconfirmed	44	hsa-mir-199a-2	26198104
20	hsa-mir-16–1	26198104	45	hsa-mir-31	unconfirmed
21	hsa-mir-638	23293055	46	hsa-mir-128–1	26198104
22	hsa-mir-21	26198104	47	hsa-mir-132	26198104
23	hsa-mir-19a	2,6041879	48	hsa-mir-15b	26166038
24	hsa-mir-203a	unconfirmed	49	hsa-mir-133a-2	unconfirmed
25	hsa-mir-23b	unconfirmed	50	hsa-mir-106a	25760076

*Note*: Gemcitabine’s Compound ID (CID) in PubChem is 60750.

## 4 Discussion

Identifying the relationships between drugs and miRNAs is helpful for the discovery of new miRNA-targeted therapies and accelerate drug discovery for complex diseases therapy. Compared with discovering drug-miRNA associations through biological experiments, predicting their associations using computational models can save time and reduce cost. In this study, we propose a new method, GNMFDMA, to infer drug-miRNA potential associations using graph Laplacian regularization collaborative non-negative matrix factorization. In GNMFDMA, we use *p*-nearest neighbors to construct sparse similarity matrix, and the new drug-miRNA association adjacency matrix is reconstructed based on the K -nearest neighbor profiles. Meanwhile, graph Laplacian regularization non-negative matrix factorization is implemented to compute the drug-miRNA association scores, which can discover the intrinsic geometrical structure from data space and extract meaningful latent features. Rigorous experimental results indicate that the performance of GNMFDMA outperforms the existing computational approaches, and can effectively reveal drug-miRNA potential associations.

Indeed, the prediction performance of GNMFDMA is still limited by some factors. Firstly, the known drug-miRNA associations are relatively sparse. With the in-depth study of drugs and miRNAs, there will be more datasets of drug-miRNA associations. Secondly, the similarity measurement in our method may not be optimal. Finally, how to effectively integrate more relevant biological information to improve prediction performance is worthy of further research.

## Data Availability

The datasets presented in this study can be found in online repositories. The names of the repository/repositories and accession number(s) can be found in the article/supplementary material.

## References

[B1] Bartel D. P. (2004). MicroRNAs: Genomics, biogenesis, mechanism, and function. Cell 116, 281–297. 10.1016/s0092-8674(04)00045-5 14744438

[B2] Batista P. J. Chang H. Y. (2013). Long noncoding RNAs: Cellular address codes in development and disease. Cell 152, 1298–1307. 10.1016/j.cell.2013.02.012 23498938 PMC3651923

[B3] Berezikov E. Cuppen E. Plasterk R. H. (2006). Approaches to microRNA discovery. Nat. Genet. 38, S2–S7. 10.1038/ng1794 16736019

[B4] Cai D. He X. Han J. Huang T. S. (2010). Graph regularized nonnegative matrix factorization for data representation. IEEE Trans. pattern analysis Mach. Intell. 33, 1548–1560. 10.1109/TPAMI.2010.231 21173440

[B5] Carninci P. Kasukawa T. Katayama S. Gough J. Frith M. Maeda N. (2005). The transcriptional landscape of the mammalian genome. Science 309, 1559–1563. 10.1126/science.1112014 16141072

[B6] Di Leva G. Cheung D. G. Croce C. M. (2015). miRNA clusters as therapeutic targets for hormone-resistant breast cancer. Expert Rev. Endocrinol. Metabolism 10, 607–617. 10.1586/17446651.2015.1099430 PMC5053393 27721895

[B7] Dixon S. J. Stockwell B. R. (2009). Identifying druggable disease-modifying gene products. Curr. Opin. Chem. Biol. 13, 549–555. 10.1016/j.cbpa.2009.08.003 19740696 PMC2787993

[B8] Dougherty T. J. Pucci M. J. (2011). Antibiotic discovery and development. Berlin, Germany: Springer Science & Business Media.

[B9] Ezzat A. Zhao P. Wu M. Li X.-L. Kwoh C.-K. (2017). Drug-target interaction prediction with graph regularized matrix factorization. IEEE/ACM Trans. Comput. Biol. Bioinforma. (TCBB) 14, 646–656. 10.1109/TCBB.2016.2530062 26890921

[B10] Facchinei F. Kanzow C. Sagratella S. (2014). Solving quasi-variational inequalities via their KKT conditions. Math. Program. 144, 369–412. 10.1007/s10107-013-0637-0

[B11] Gottlieb A. Stein G. Y. Ruppin E. Sharan R. (2011). Predict: A method for inferring novel drug indications with application to personalized medicine. Mol. Syst. Biol. 7, 496. 10.1038/msb.2011.26 21654673 PMC3159979

[B12] Guan N.-N. Sun Y.-Z. Ming Z. Li J.-Q. Chen X. (2018). Prediction of potential small molecule-associated microRNAs using graphlet interaction. Front. Pharmacol. 9, 1152. 10.3389/fphar.2018.01152 30374302 PMC6196296

[B13] Hattori M. Okuno Y. Goto S. Kanehisa M. (2003). Development of a chemical structure comparison method for integrated analysis of chemical and genomic information in the metabolic pathways. J. Am. Chem. Soc. 125, 11853–11865. 10.1021/ja036030u 14505407

[B14] He L. Hannon G. J. (2004). MicroRNAs: Small RNAs with a big role in gene regulation. Nat. Rev. Genet. 5, 522–531. 10.1038/nrg1379 15211354

[B15] Hopkins A. L. Groom C. R. (2002). The druggable genome. Nat. Rev. Drug Discov. 1, 727–730. 10.1038/nrd892 12209152

[B16] Huang Y.-A. Chan K. C. You Z.-H. Hu P. Wang L. Huang Z.-A. (2021). Predicting microRNA–disease associations from lncRNA–microRNA interactions via multiview multitask learning. Briefings Bioinforma. 22, bbaa133. 10.1093/bib/bbaa133 32633319

[B17] Huang Y.-a. Hu P. Chan K. C. You Z.-H. (2020). Graph convolution for predicting associations between miRNA and drug resistance. Bioinformatics 36, 851–858. 10.1093/bioinformatics/btz621 31397851

[B18] Huang Y.-A. You Z.-H. Chen X. (2018). A systematic prediction of drug-target interactions using molecular fingerprints and protein sequences. Curr. Protein Peptide Sci. 19, 468–478. 10.2174/1389203718666161122103057 27875970

[B19] Huang Y.-A. You Z.-H. Li X. Chen X. Hu P. Li S. (2016). Construction of reliable protein–protein interaction networks using weighted sparse representation based classifier with pseudo substitution matrix representation features. Neurocomputing 218, 131–138. 10.1016/j.neucom.2016.08.063

[B20] Huang Y. You Z. Chen X. Huang Z. Zhang S. Yan G. (2017). Prediction of microbe-disease association from the integration of neighbor and graph with collaborative recommendation model. J. Transl. Med. 15, 209. 10.1186/s12967-017-1304-7 29037244 PMC5644104

[B21] Jiang Q. Wang Y. Hao Y. Juan L. Teng M. Zhang X. (2008). miR2Disease: a manually curated database for microRNA deregulation in human disease. Nucleic Acids Res. 37, D98–D104. 10.1093/nar/gkn714 18927107 PMC2686559

[B22] Jiang W. Chen X. Liao M. Li W. Lian B. Wang L. (2012). Identification of links between small molecules and miRNAs in human cancers based on transcriptional responses. Sci. Rep. 2, 282. 10.1038/srep00282 22355792 PMC3282946

[B23] Jiang X. Hu X. Xu W. (2015). Microbiome data representation by joint nonnegative matrix factorization with laplacian regularization. IEEE/ACM Trans. Comput. Biol. Bioinforma. 14, 353–359. 10.1109/TCBB.2015.2440261 28368813

[B24] Knox C. Law V. Jewison T. Liu P. Ly S. Frolkis A. (2010). DrugBank 3.0: A comprehensive resource for ‘omics’ research on drugs. Nucleic Acids Res. 39, D1035–D1041. 10.1093/nar/gkq1126 21059682 PMC3013709

[B25] Kozomara A. Birgaoanu M. Griffiths-Jones S. (2018). miRBase: from microRNA sequences to function. Nucleic Acids Res. 47, D155–D162. 10.1093/nar/gky1141 PMC6323917 30423142

[B26] Krzyzosiak A. Sigurdardottir A. Luh L. Carrara M. Das I. Schneider K. (2018). Target-based discovery of an inhibitor of the regulatory phosphatase PPP1R15B. Cell 174, 1216–1228. 10.1016/j.cell.2018.06.030 30057111 PMC6108835

[B27] Lanford R. E. Hildebrandt-Eriksen E. S. Petri A. Persson R. Lindow M. Munk M. E. (2010). Therapeutic silencing of microRNA-122 in primates with chronic hepatitis C virus infection. Science 327, 198–201. 10.1126/science.1178178 19965718 PMC3436126

[B28] Lee D. D. Seung H. S. (1999). Learning the parts of objects by non-negative matrix factorization. Nature 401, 788–791. 10.1038/44565 10548103

[B29] Li J. Lei K. Wu Z. Li W. Liu G. Liu J. (2016). Network-based identification of microRNAs as potential pharmacogenomic biomarkers for anticancer drugs. Oncotarget 7, 45584–45596. 10.18632/oncotarget.10052 27329603 PMC5216744

[B30] Li L.-Q. Li X.-L. Wang L. Du W.-J. Guo R. Liang H.-H. (2012). Matrine inhibits breast cancer growth via miR-21/PTEN/Akt pathway in MCF-7 cells. Cell. Physiology Biochem. 30, 631–641. 10.1159/000341444 22832383

[B31] Li J. Cui G. Dong Y. (2016). Graph regularized non-negative low-rank matrix factorization for image clustering. IEEE Trans. Cybern. 47, 3840–3853. 10.1109/TCYB.2016.2585355 27448379

[B32] Li Y. Vanden T. G. Kong D. Wang Z. Ali S. Philip P. A. (2009). Up-regulation of miR-200 and let-7 by natural agents leads to the reversal of epithelial-to-mesenchymal transition in gemcitabine-resistant pancreatic cancer cells. Cancer Res. 69, 6704–6712. 10.1158/0008-5472.CAN-09-1298 19654291 PMC2727571

[B33] Liu X. Wang S. Meng F. Wang J. Zhang Y. Dai E. (2012). SM2miR: A database of the experimentally validated small molecules’ effects on microRNA expression. Bioinformatics 29, 409–411. 10.1093/bioinformatics/bts698 23220571

[B34] Liu X. Zhai D. Zhao D. Zhai G. Gao W. (2014). Progressive image denoising through hybrid graph laplacian regularization: A unified framework. IEEE Trans. Image Process. 23, 1491–1503. 10.1109/TIP.2014.2303638 24565791

[B35] Lu M. Zhang Q. Deng M. Miao J. Guo Y. Gao W. (2008). An analysis of human microRNA and disease associations. PloS One 3, e3420. 10.1371/journal.pone.0003420 18923704 PMC2559869

[B36] Lv S. Li Y. Wang Q. Ning S. Huang T. Wang P. (2011). A novel method to quantify gene set functional association based on gene ontology. J. R. Soc. Interface 9, 1063–1072. 10.1098/rsif.2011.0551 21998111 PMC3306647

[B37] Lv Y. Wang S. Meng F. Yang L. Wang Z. Wang J. (2015). Identifying novel associations between small molecules and miRNAs based on integrated molecular networks. Bioinformatics 31, 3638–3644. 10.1093/bioinformatics/btv417 26198104

[B38] Peng L. Wang F. Wang Z. Tan J. Huang L. Tian X. (2022). Cell–cell communication inference and analysis in the tumour microenvironments from single-cell transcriptomics: Data resources and computational strategies. Briefings Bioinforma. 23, bbac234. 10.1093/bib/bbac234 35753695

[B39] Qu J. Chen X. Sun Y.-Z. Li J.-Q. Ming Z. (2018). Inferring potential small molecule–miRNA association based on triple layer heterogeneous network. J. Cheminformatics 10, 30. 10.1186/s13321-018-0284-9 PMC6020102 29943160

[B40] Ruepp A. Kowarsch A. Schmidl D. Buggenthin F. Brauner B. Dunger I. (2010). PhenomiR: A knowledgebase for microRNA expression in diseases and biological processes. Genome Biol. 11, R6. 10.1186/gb-2010-11-1-r6 20089154 PMC2847718

[B41] Rupaimoole R. Slack F. J. (2017). MicroRNA therapeutics: Towards a new era for the management of cancer and other diseases. Nat. Rev. Drug Discov. 16, 203–222. 10.1038/nrd.2016.246 28209991

[B42] Santos R. Ursu O. Gaulton A. Bento A. P. Donadi R. S. Bologa C. G. (2017). A comprehensive map of molecular drug targets. Nat. Rev. Drug Discov. 16, 19–34. 10.1038/nrd.2016.230 27910877 PMC6314433

[B43] Shen L. Liu F. Huang L. Liu G. Zhou L. Peng L. (2022). VDA-RWLRLS: An anti-SARS-CoV-2 drug prioritizing framework combining an unbalanced bi-random walk and Laplacian regularized least squares. Comput. Biol. Med. 140, 105119. 10.1016/j.compbiomed.2021.105119 PMC8664497 34902608

[B44] Sui W. Lin H. Peng W. Huang Y. Chen J. Zhang Y. (2013). Molecular dysfunctions in acute rejection after renal transplantation revealed by integrated analysis of transcription factor, microRNA and long noncoding RNA. Genomics 102, 310–322. 10.1016/j.ygeno.2013.05.002 23684794

[B45] Wang L. Chen X. Qu J. Sun Y.-Z. Li J.-Q. (2019). Rfsmma: A newcomputational model to identify and prioritize potential small molecule–MiRNA associations. J. Chem. Inf. Model. 59, 1668–1679. 10.1021/acs.jcim.9b00129 30840454

[B46] Wang L. You Z.-H. Chen X. Li Y.-M. Dong Y.-N. Li L.-P. (2019). Lmtrda: Using logistic model tree to predict MiRNA-disease associations by fusing multi-source information of sequences and similarities. PLoS Comput. Biol. 15, e1006865. 10.1371/journal.pcbi.1006865 30917115 PMC6464243

[B47] Wang L. You Z.-H. Chen X. Yan X. Liu G. Zhang W. (2018). Rfdt: A rotation forest-based predictor for predicting drug-target interactions using drug structure and protein sequence information. Curr. Protein Peptide Sci. 19, 445–454. 10.2174/1389203718666161114111656 27842479

[B48] Wang L. You Z.-H. Zhou X. Yan X. Li H.-Y. Huang Y.-A. (2021). Nmfcda: Combining randomization-based neural network with non-negative matrix factorization for predicting CircRNA-disease association. Appl. Soft Comput. 110, 107629. 10.1016/j.asoc.2021.107629

[B49] Wang M. Xie X.-J. You Z.-H. Wong L. Li L.-P. Chen Z.-H. (2022). Combining K nearest neighbor with nonnegative matrix factorization for predicting circrna-disease associations. IEEE/ACM Trans. Comput. Biol. Bioinforma. 2022, 1–10. 10.1109/TCBB.2022.3180903 35675235

[B50] Wang L. You Z.-H. Wang L. Li L.-P. Zheng K. (2021). Ldgrnmf: LncRNA-disease associations prediction based on graph regularized non-negative matrix factorization. Neurocomputing 424, 236–245. 10.1016/j.neucom.2020.02.062

[B51] Wang M. Lei L.-L. He W. Ding D. (2022). Spcmlmi: A structural perturbation-based matrix completion method to predict LncRNA-MiRNA interactions. Front. Genet. 13, 1032428. 10.3389/fgene.2022.1032428 36457751 PMC9705354

[B52] Wang Y. Xiao J. Suzek T. O. Zhang J. Wang J. Bryant S. H. (2009). PubChem: A public information system for analyzing bioactivities of small molecules. Nucleic Acids Res. 37, W623–W633. 10.1093/nar/gkp456 19498078 PMC2703903

[B53] Wheeler H. E. Maitland M. L. Dolan M. E. Cox N. J. Ratain M. J. (2013). Cancer pharmacogenomics: Strategies and challenges. Nat. Rev. Genet. 14, 23–34. 10.1038/nrg3352 23183705 PMC3668552

[B54] Wightman B. Ha I. Ruvkun G. (1993). Posttranscriptional regulation of the heterochronic gene lin-14 by lin-4 mediates temporal pattern formation in *C. Elegans* . Cell 75, 855–862. 10.1016/0092-8674(93)90530-4 8252622

[B55] Yao J. Li Z. Wang X. Xu P. Zhao L. Qian J. (2016). MiR-125a regulates chemo-sensitivity to gemcitabine in human pancreatic cancer cells through targeting A20. Acta Biochimica Biophysica Sinica 48, 202–208. 10.1093/abbs/gmv129 26758190

[B56] Yin J. Chen X. Wang C.-C. Zhao Y. Sun Y.-Z. (2019). Prediction of small molecule-microRNA associations by sparse learning and heterogeneous graph inference. Mol. Pharm. 16, 3157–3166. 10.1021/acs.molpharmaceut.9b00384 31136190

[B57] You Z.-H. Huang Z.-A. Zhu Z. Yan G.-Y. Li Z.-W. Wen Z. (2017). Pbmda: A novel and effective path-based computational model for miRNA-disease association prediction. PLoS Comput. Biol. 13, e1005455. 10.1371/journal.pcbi.1005455 28339468 PMC5384769

[B58] You Z.-H. Lei Y.-K. Gui J. Huang D.-S. Zhou X. (2010). Using manifold embedding for assessing and predicting protein interactions from high-throughput experimental data. Bioinformatics 26, 2744–2751. 10.1093/bioinformatics/btq510 20817744 PMC3025743

